# A Caputo–Fabrizio Fractional-Order Model of HIV/AIDS with a Treatment Compartment: Sensitivity Analysis and Optimal Control Strategies

**DOI:** 10.3390/e23050610

**Published:** 2021-05-14

**Authors:** Hua Wang, Hadi Jahanshahi, Miao-Kun Wang, Stelios Bekiros, Jinping Liu, Ayman A. Aly

**Affiliations:** 1 School of Mathematics and Statistics, Changsha University of Science and Technology, Changsha 410114, China; hncswhua@csust.edu.cn; 2 Department of Mechanical Engineering, University of Manitoba, Winnipeg, MB R3T 5V6, Canada; Jahanshahi.hadi90@gmail.com; 3 Department of Mathematics, Huzhou University, Huzhou 313000, China; 4 Department of Banking and Finance, FEMA, University of Malta, MSD 2080 Msida, Malta; 5 European University Institute, Department of Economics, Via delle Fontanelle, 18, I-50014 Florence, Italy; 6 Hunan Provincial Key Laboratory of Intelligent Computing and Language Information Processing, Hunan Normal University, Changsha 410081, China; Ljp202518@163.com; 7 Department of Mechanical Engineering, College of Engineering, Taif University, P.O. Box 11099, Taif 21944, Saudi Arabia; aymanaly@tu.edu.sa

**Keywords:** HIV model, treatment compartment, Caputo–Fabrizio fractional, optimal control, sensitivity analysis

## Abstract

Although most of the early research studies on fractional-order systems were based on the Caputo or Riemann–Liouville fractional-order derivatives, it has recently been proven that these methods have some drawbacks. For instance, kernels of these methods have a singularity that occurs at the endpoint of an interval of definition. Thus, to overcome this issue, several new definitions of fractional derivatives have been introduced. The Caputo–Fabrizio fractional order is one of these nonsingular definitions. This paper is concerned with the analyses and design of an optimal control strategy for a Caputo–Fabrizio fractional-order model of the HIV/AIDS epidemic. The Caputo–Fabrizio fractional-order model of HIV/AIDS is considered to prevent the singularity problem, which is a real concern in the modeling of real-world systems and phenomena. Firstly, in order to find out how the population of each compartment can be controlled, sensitivity analyses were conducted. Based on the sensitivity analyses, the most effective agents in disease transmission and prevalence were selected as control inputs. In this way, a modified Caputo–Fabrizio fractional-order model of the HIV/AIDS epidemic is proposed. By changing the contact rate of susceptible and infectious people, the atraumatic restorative treatment rate of the treated compartment individuals, and the sexual habits of susceptible people, optimal control was designed. Lastly, simulation results that demonstrate the appropriate performance of the Caputo–Fabrizio fractional-order model and proposed control scheme are illustrated.

## 1. Introduction

Over the past several years, various studies have been carried out to construct an appropriate mathematical model for various disease dynamics, including those of tuberculosis, malaria, and HIV [1,2,3,4,5]. Mathematical modeling of diseases plays an important role in profound understanding of the system for the purpose of disease control due to the fact that it enables long- and short-term prediction of disease incidence [6,7,8,9,10,11]. Since the study by [12] on the modeling of diseases, which was a breakthrough in this area, dynamical systems approaches have been used for a wide variety of diseases. So far, theoretical epidemiology has resulted in numerous remarkable technical and conceptual developments. The goal of this field of study is not only to analyze and anticipate the spread of various diseases but also to control it as effectively as possible.

HIV is one of the most hazardous threats to human health. The virus occupies the T cells in the early stage of HIV. By entering the T cells, HIV viruses which previously could not replicate by themselves easily build a virus factory [13]. In the last step of the infection, HIV debilitates the human immune system and brings about acquired immune deficiency syndrome (AIDS) [14]. An impaired immune system cannot overcome infectious diseases, and this situation sometimes causes death. Thus, so far, many research studies have focused on the control of HIV/AIDS infection to find a way to prevent it from spreading.

Despite the long history of fractional calculus, its applications are only a new subject of interest. Fractional calculus has recently been utilized in various fields of study [15,16,17,18,19,20,21,22,23,24,25,26,27,28,29,30,31,32]. Also, the modeling of HIV using fractional differential equations has started to attract some research attention. For instance, a fractional-order model of HIV infection of T cells was introduced by Ding and Ye [33]. They also investigated the stability of equilibrium via detailed analysis. Fractional-order differential models of the dynamics of HIV infection of CD4+ T cells and the dynamics of the tumor–immune system were proposed by Rihan [34]. A fractional-order model for the three stages of HIV epidemics, encompassing drug resistance, was introduced by Pinto and Carvalho [35]. Dutta et al. conducted an analysis on the fractional-order deterministic HIV/AIDS model during drug therapy treatment [36].

Although most of the early research studies on fractional-order systems were based on the Caputo or Riemann–Liouville fractional-order derivative, it has been proven that these methods have some drawbacks. For instance, kernels of these methods have a singularity that occurs at the endpoint of an interval of definition [37,38,39]. Thus, to overcome this issue, several new definitions of fractional derivatives have been introduced [40,41,42,43,44,45]. The basic differences among these derivatives are their different kernels, which should be chosen to satisfy the requirements of various systems. The main differences between the Caputo–Fabrizio (CF) and the Caputo fractional derivative are that the CF derivative is obtained using an exponential decay law, but the Caputo derivative is based on a power law [38,46].

Several research studies have demonstrated the applications of the new fractional derivatives to practical systems. For instance, the Atangana–Baleanu and CF fractional derivatives for chaotic systems and fractional delay differential equations were compared by Atangana et al. [47,48]. They showed that the Atangana–Baleanu fractional results in noisy information because of its specific memory properties. On the other hand, the CF fractional derivative yields less noise than the Atangana–Baleanu fractional derivative. Moore et al. [49] considered HIV/AIDS with an antiretroviral treatment compartment and proposed a CF fractional equation for this system. They demonstrated the effectiveness of the CF derivative for modelling HIV/AIDS.

So far, various schemes have been introduced to control nonlinear systems [50,51,52,53,54,55,56,57,58]. As well, for HIV–immune systems, as nonlinear systems, there are a wide variety of controllers in the literature, including a fuzzy discrete event system approach [59,60], feedback control [61,62], sliding mode control [14], and optimal control [63,64,65,66,67]. Among these controllers, optimal control theory is an effective tool in disease control because it presents appropriate preventive and treatment strategies by considering various factors in the optimization function. Hence, optimal control has attracted much attention in this research area.

To the best of our knowledge, no study has designed a controller for the CF fractional model of HIV/AIDS. The CF fractional is a new fractional definition that is very beneficial to the modeling of real-world problems [68]. Moreover, although control of HIV/AIDS has been studied in the literature, there are still other meaningful behaviors of these systems during various strategies which need to be further understood. Hence, in this study, the dynamics of a CF fractional model for HIV/AIDS are studied. Then, an optimal controller is designed for the system, and various strategies are precisely investigated.

The rest of this study is presented as follows: Firstly, a CF fractional model for HIV/AIDS with a treatment compartment is studied in Section 2. In Section 3, the equilibrium point of the model and its stability are investigated. In Section 4, sensitivity analysis for the system is performed. The general formulation of a Fractional Optimal Control Problem (FOCP) and the necessary conditions for its optimality are described in Section 5. Fractional optimal control of the HIV/AIDS model is designed in Section 6. Afterward, in Section 7, the simulation results of several control strategies, such as control using prevention, treatment, and changing of sexual habits, are demonstrated. Lastly, the conclusions are presented in Section 8.

## 2. A CF Fractional Model of HIV/AIDS with a Treatment Compartment

A CF fractional model of HIV/AIDS with a treatment compartment was considered in the current study. The non-dimensional model is written as follows [49]:

(1) D0CFtα11S(t)=Λ−βI(t)S(t)−μ1S(t)−dS(t)D0CFtα22I(t)=βI(t)S(t)+α1T(t)−dI(t)−k1I(t)−k2I(t)D0CFtα33A(t)=k1I(t)−(δ1+d)A(t)+α2T(t)D0CFtα44T(t)=k2I(t)−α1T(t)−(α2+d+δ2)T(t)D0CFtα55R(t)=μ1S(t)−dR(t)

The initial conditions are

(2) $$S\left(0\right)=S_{0},\;I\left(0\right)=I_{0},A\left(0\right)=A_{0},\;T\left(0\right)=T_{0},R\left(0\right)=R_{0},$$

where states are defined as follows: S(t) and I(t) denote the number of susceptible patients and the number of HIV-positive individuals who are infectious, respectively; A(t) is the number of individuals for whom the treatment is not effective or who are not receiving ART treatment. The total number of individuals being treated with ART and for whom the treatment is effective is represented by T(t). R(t) indicates the individuals who have changed their sexual habits and who are thus immune to HIV infection by sexual contact. In addition, Λ is the recruitment rate of susceptible individuals into the population. β is the contact rate between susceptible and infectious individuals. $\mu_{1}$ is the rate at which susceptible individuals change their sexual habits, and $\alpha_{1}$ is the rate at which treated individuals leave the treated compartment and return to the infectious class. $\delta_{1}$ and $\delta_{2}$ are the disease-induced death rates for individuals in compartments A(t) and T(t), respectively. $k_{1}$ is the rate at which members leave the infectious compartment and become individuals with full-blown AIDS. $k_{2}$ represents the rate at which individuals with HIV receive treatment. Finally, $\alpha_{2}$ is defined as the rate at which treated individuals leave the treated class and enter the AIDS compartment, A(t). This model is non-dimensional.

## 3. Equilibrium Point of the Model

In this section, the equilibrium point of the fractional model of HIV/AIDS is obtained. From [49], the equilibrium point of the system is as follows:

(3) $$E_{df}=\left(\begin{array}{ccc} \frac{\Lambda }{\left(\mu_{1}+d\right)} & 0 & \begin{array}{ccc} 0 & 0 & \frac{\Lambda \mu_{1}}{d\left(\mu_{1}+d\right)} \end{array} \end{array}\right)$$

where $E_{df}$ is the disease-free equilibrium point, and the endemic equilibrium point is

(4) $$E_{e}=\left(\begin{array}{ccccc} S^{*} & I^{*} & A^{*} & T^{*} & R^{*} \end{array}\right)$$

Also, $R^{0}$ is the basic reproduction number, which can be calculated using the next-generation matrix method [69,70], and it is as follows:

(5) $$R^{0}=\frac{\beta \Lambda \left(\alpha_{1}+d+\delta_{2}+\alpha_{2}\right)}{\left(\mu_{1}+d\right)\left(d+k_{1}+k_{2}\right)\left(\alpha_{1}+d+\delta_{2}+\alpha_{2}\right)-\alpha_{1}k_{2}}$$

It was previously proven that the disease-free equilibrium point of the CF fractional model of HIV/AIDS with treatment compartment $E_{df}$ is asymptotically stable [49].

## 4. Sensitivity Analysis

In this section, sensitivity analysis of the basic reproduction number and the endemic equilibrium points is conducted. These analyses reveal the factors that have effects on the populations of different compartments. Using this analysis, we can find out how the population of each compartment can be controlled in order to control disease transmission and prevalence. The system parameters that were used in these analyses are given in Table 1 [49].

The following definition delineates the sensitivity analysis procedure that was carried out in the current study.

**Definition** **1.** *The normalized forward sensitivity index of a variable h that depends on parameter* l *is defined as* $\Upsilon_{l}^{h}=\frac{\delta h}{\delta l}\times \frac{h}{l}$ *.*

Herein, we calculate the sensitivity indices of $R^{0}$ to all parameters of the model by $\Upsilon_{l}^{R^{0}}=\frac{\delta R^{0}}{\delta l}\times \frac{R^{0}}{l}$, where *l* indicates the parameters of the model.

The reproductive number affects the initial transmission of the disease. Furthermore, the disease prevalence is highly related to the endemic equilibrium point. Therefore, the sensitivity of the reproductive number to the system parameters was calculated, and the results are given in Table 2; the sensitivity indices of the state variables at the endemic equilibrium point to the model parameters are given in Table 3.

The sensitivity index values for the endemic equilibrium point and reproduction number were calculated using MATLAB and are given in Table 2 and Table 3. The sensitivity indices in Table 3 show that the state variables of the endemic equilibrium point that are important for us are highly affected by three parameters. The first parameter is β, for which the sensitivity index is −1 for the first state variable of the endemic equilibrium point, and this value is −0.8762 for the second and third state variables. The second parameter that has a large sensitivity index is $\alpha_{1},$ for which the sensitivity index for the first state variable is 0.7231, that for the second state variable is −0.0894, and that for the third state variable is −0.4970. Finally, the third variable is $\mu_{1}$; its sensitivity index values for the first, second, and third state variables are −0.7382, 0.6213, and 0.6213, respectively.

The results of the sensitivity analyses show that the three aforementioned parameters may be effective in controlling the disease. Therefore, one control effort is to change the contact rate between susceptible and infectious people (β). The second control effort is to change the rate at which people in the treated compartment return to the infectious class ($\alpha_{1}$). Finally, the last control effort is to change the rate of changes in sexual habits of individuals in the susceptible class ($\mu_{1}$).

## 5. Necessary Conditions for Optimality of an FOCP

This section describes the general formulation of an FOCP and the necessary conditions for its optimality. An FOCP can be defined as follows:

(6) $$J\left(u\right)=\int_{0}^{t_{f}}L\left(t,x,u\right)dt$$

This is subject to the dynamic constraint

(7) D0CFtαx(t)=f(t,x,u)

with initial condition $x\left(0\right)=x_{0}$. Here, x(t) and u(t) are state and control vectors, respectively. L and f are differentiable functions, and 0<α≤1.

**Theorem** **1.** *We define a Hamiltonian as follows:*

(8) H(t,x,u,λ)=L(t,x,u)+λ∗f(t,x,u)

*where* $\lambda \in C^{1}\left[0.t_{f}\right]$ *is a function. If*λ, x, u satisfy the equations

(9) D0CFtαx(t)=∂H(t, x(t), u(t), λ(t))∂λDtCFtfαλ(t)=∂H(t, x(t),u(t),λ(t))∂x∂H(t, x(t),u(t),λ(t))∂u=0λ(tf)=0

*then* *(*x,u) *is the minimizer of Equation (6).*

**Proof.**  Substituting Equation (8) into Equation (6) results in the following equation:

(10) $$J\left(u\right)=\int_{0}^{t_{f}}\left(H\left(t,x,u,\lambda \right)-\lambda .f\left(t,x,u\right)\right)dt$$

The necessary condition for the optimality of an FOCP is Equation (11):

(11) δJ(u)=0

Therefore, to obtain the optimal control laws, by taking the variation of Equation (10), the right side of Equation (10) is calculated:

(12) δJ(u)=∫0tf[δx∂H∂x+δu∂H∂u+δλ∂H∂λ−δλ.D0CFtαx(t)−λ.δ(D0CFtαx(t))]dt

where δx, δu, and δλ are the variations of x, u, and λ, respectively. It can be calculated that [71]

(13) ∫0tfλ(t).δ(D0CFtαx(t))dt=(ItCFtf1−αλ(t))−∫0tfδx.(D0CFtαλ(t))dt

Now, by substituting Equation (13) into Equation (12), we have

(14) δJ(u)=∫0tf[δx[∂H∂x−D0CFtαλ(t)]+δu[∂H∂u]+δλ[∂H∂λ−D0CFtαx(t)]]dt+(ItCFtf1−αλ(t))δx|t=tf.

By taking Equation (14) into consideration, it can be concluded that the coefficients of δx, δu, and δλ must be equal to zero, leading to the following equations:

(15) D0CFtαx(t)=∂H(t,x(t),u(t),λ(t))∂λDtCFtfαλ(t)=∂H(t,x(t),u(t),λ(t))∂x∂H(t,x(t),u(t),λ(t))∂u=0ItCFtf1−αλ(t)|t=tf=0

Since λ(t) is a continuous function, it can be concluded that

(16) ItCFtf1−αλ(t)|t=tf=λ(tf)

Also, it has been proven that the following equations hold [72]. □

**Lemma** **1.** *The following equations hold:*

(17) DtCFtfαλ(t)=∂H(t,x(t),u(t),λ(t))∂x

(18) D0CFtαλ(tf−t)=∂H(tf−t,x(tf−t),u(tf−t),λ(tf−t))∂x

*where* 0<α≤1*.*

**Proof.**  The CF fractional derivative is defined as follows [37]:

(19) D0CFtαf(t)=11−α∫0tf′(x)exp(−αt−x1−α)dx, t>0

It is obvious that

(20) DtCFtfαλ(t)=11−α∫ttfλ′(x)exp(−αx−t1−α)dx

Now, replacing t by $t_{f}-t$ in Equation (20) gives

(21) Dtf−tCFtfαλ(tf−t)=11−α∫tf−ttfλ′(x)exp(−αx−tf+t1−α)dx

By defining a new variable as $w=t_{f}-x$, Equation (21) can be written in the following form:

(22) Dtf−tCFtfαλ(tf−t)=11−α∫t0λ′(tf−w)exp(−αt−w1−α)(−dw)=−11−α∫0t(λ(tf−w))’exp(−αt−w1−α)(dw)=D0CFtαλ(tf−t)

Therefore, the optimality conditions are as follows:

(23) D0CFtαx(t)=∂H(t,x(t),u(t),λ(t))∂λD0CFtαλ(t)=∂H(tf−t,x(tf−t),u(tf−t),λ(tf−t)) ∂x∂H(t,x(t),u(t),λ(t))∂u=0

□

## 6. Fractional Optimal Control of the HIV/AIDS Model

In this section, using sensitivity analyses, the fractional model of HIV/AIDS proposed by [49] is modified. The proposed model was developed in order to reduce infection using control via condom use, $u_{1}$; optimization of ART treatment via control $u_{2};$ and changing individual habits in order to reduce infection by means of control $u_{3}$. Optimal control was implemented in order to find the optimal control actions for the modified model developed in this research. The modified proposed model is written as follows:

(24) D0CFtα11S(t)=Λ−β(1−ε1u1(t))I(t)S(t)−u3(t)S(t)−dS(t)D0CFtα22I(t)=β(1−ε1u1(t))I(t)S(t)+ε2u2(t)T(t)−dI(t)−k1I(t)−k2I(t)D0CFtα33A(t)=k1I(t)−(δ1+d)A(t)+α2T(t)D0CFtα44T(t)=k2I(t)−ε2u2(t)T(t)−(α2+d+δ2)T(t)D0CFtα55R(t)=u3(t)S(t)−dR(t)

The initial values of the states are

(25) $$S\left(0\right)=S_{0},\;I\left(0\right)=I_{0},A\left(0\right)=A_{0},\;T\left(0\right)=T_{0},R\left(0\right)=R_{0}.$$

In the proposed model, it was assumed that $\alpha_{11}=\alpha_{22}=\alpha_{33}=\alpha_{44}=\alpha_{55}=\alpha$.

As mentioned, $u_{1}$ is control via condom use, so using this control input, the rate of contact between the susceptible population and the infectious population can be reduced; $\varepsilon_{1}\in \left(0,1\right)$ measures the effectiveness of condom use. $u_{2}$ is control of the ART treatment rate of the population in the treated compartment; $\varepsilon_{2}\epsilon \left(0,1\right]$ is the effectiveness of ART treatment in increasing the level of $CD4^{+}$ T cells. Lastly, $u_{3}$ is the rate at which susceptible people change their sexual habits per unit time.

(26) $$J\left(u\right)=\int_{0}^{t_{f}}\left[AS\left(t\right)+BT\left(t\right)+\frac{C_{1}}{2}u_{1}^{2}\left(t\right)+\frac{C_{2}}{2}u_{2}^{2}\left(t\right)+\frac{C_{3}}{2}u_{3}^{2}\left(t\right)\right]dt$$

Subject to the modified model in Equation (24), the Hamiltonian of the problem is defined as follows:

(27) $$H=AS\left(t\right)+BT\left(t\right)+\frac{C_{1}}{2}u_{1}^{2}\left(t\right)+\frac{C_{2}}{2}u_{2}^{2}\left(t\right)+\frac{C_{3}}{2}u_{3}^{2}\left(t\right)+\lambda_{1}(\Lambda -\beta (1-\;\varepsilon_{1}u_{1}\left(t\right))I\left(t\right)S\left(t\right)-u_{3}\left(t\right)S\left(t\right)-dS\left(t\right))+\lambda_{2}(\beta \left(1-\varepsilon_{1}u_{1}\left(t\right)\right)I\left(t\right)S\left(t\right)+\;\varepsilon_{2}u_{2}\left(t\right)T\left(t\right)-dI\left(t\right)-k_{1}I\left(t\right)-k_{2}I\left(t\right))+\lambda_{3}\left(k_{1}I\left(t\right)-\left(\delta_{1}+d\right)A\left(t\right)+\alpha_{2}T\left(t\right)\right)+\;\lambda_{4}(k_{2}I\left(t\right)-\varepsilon_{2}u_{2}\left(t\right)T\left(t\right)-\left(\alpha_{2}+d+\delta_{2}\right)T\left(t\right)+\lambda_{5}\left(u_{3}\left(t\right)S\left(t\right)-dR\left(t\right)\right)$$

As demonstrated in Equation (23), the necessary conditions for the optimality of (26) are:

(28) D0CFtαS(t)=Λ−β(1−ε1u1(t))I(t)S(t)−u3(t)S(t)−dS(t)D0CFtαI(t)=β(1−ε1u1(t))I(t)S(t)+ε2u2(t)T(t)−dI(t)−k1I(t)−k2I(t)D0CFtαA(t)=k1I(t)−(δ1+d)A(t)+α2T(t)D0CFtαT(t)=k2I(t)−ε2u2(t)T(t)−(α2+d+δ2)T(t)D0CFtαR(t)=u3(t)S(t)−dR(t)

For co-states, we have:

(29) D0CFtαλ1(tf−t)=A+β(1−ε1u1(tf−t))I(tf−t)(λ2(tf−t)−λ1(tf−t))−(u3(tf−t)+d)λ1(tf−t)D0CFtαλ2(tf−t)=β(1−ε1u1(tf−t))S(tf−t)(λ2(tf−t)−λ1(tf−t))−(d+k1+k2)λ2(tf−t)+k1λ3(tf−t)+k2λ4(tf−t)D0CFtαλ3(tf−t)=−(δ1+d)λ3(tf−t)D0CFtαλ4(tf−t)=−ε2u2(t)λ2(tf−t)+α2λ3(tf−t)+(u2(tf−t)−(α2+d+δ2))λ4(tf−t)+BD0CFtαλ5(tf−t)=−dλ5(tf−t)

Further,

(30) $$\frac{\partial H}{\partial u_{1}}=C_{1}u_{1}\left(t\right)+\lambda_{2}\left(t\right)\varepsilon_{1}\beta I\left(t\right)S\left(t\right)-\lambda_{1}\left(t\right)\varepsilon_{1}\beta I\left(t\right)S\left(t\right)=0\;\frac{\partial H}{\partial u_{2}}=C_{2}u_{2}\left(t\right)+\varepsilon_{2}\lambda_{2}\left(t\right)T\left(t\right)-\varepsilon_{2}\lambda_{4}\left(t\right)T\left(t\right)=0\;\frac{\partial H}{\partial u_{3}}=C_{3}u_{3}\left(t\right)-\lambda_{1}\left(t\right)S\left(t\right)+\lambda_{5}\left(t\right)S\left(t\right)=0$$

Also, the Lagrange multiplier vector must satisfy Equation (29). Using Equation (30), the optimal controls are obtained as follows:

$$u_{1}^{\prime }\left(t\right)=\frac{\left(\lambda_{2}\left(t\right)\varepsilon_{1}\beta I\left(t\right)S\left(t\right)-\lambda_{1}\left(t\right)\varepsilon_{1}\beta I\left(t\right)S\left(t\right)\right)}{C_{1}}\;u_{2}^{\prime }\left(t\right)=\frac{\varepsilon_{2}\lambda_{4}\left(t\right)T\left(t\right)-\varepsilon_{2}\lambda_{2}\left(t\right)T\left(t\right)}{C_{2}}\;u_{3}^{\prime }\left(t\right)=\frac{\lambda_{1}\left(t\right)S\left(t\right)-\lambda_{5}\left(t\right)S\left(t\right)}{C_{3}}$$

Then, the optimal controls are defined as follows:

$$u_{1}^{*}\left(t\right)=\{\begin{array}{cc} 0 & if\;u_{1}^{\prime }\left(t\right)<0\\ u_{1}^{\prime }\left(t\right) & if\;0<u_{1}^{\prime }\left(t\right)<1\\ 1 & if\;u_{1}^{\prime }\left(t\right)>1 \end{array}\;u_{2}^{*}\left(t\right)=\{\begin{array}{cc} 0 & if\;u_{2}^{\prime }\left(t\right)<0\\ u_{2}^{\prime }\left(t\right) & if\;0<u_{2}^{\prime }\left(t\right)<1\\ 1 & if\;u_{2}^{\prime }\left(t\right)>1 \end{array}\;u_{3}^{*}\left(t\right)=\{\begin{array}{cc} 0 & if\;u_{3}^{\prime }\left(t\right)<0\\ u_{3}^{\prime }\left(t\right) & if\;0<u_{3}^{\prime }\left(t\right)<1\\ 1 & if\;u_{3}^{\prime }\left(t\right)>1 \end{array}$$

## 7. Numerical Simulations

Herein, through numerical simulations, the control of the fractional model of HIV/AIDS was studied. The proposed optimal control was implemented to minimize the number of susceptible people, the population of the treated infectious people compartment, and the cost of control efforts. In this paper, a three-step fractional Adams–Bashforth scheme was implemented to calculate numerical solutions for the CF fractional differential equations. The Adams–Bashforth is described first; this method was applied to the state and co-state fractional equations, Equations (22) and (23). The iterative scheme was used for solving the system dynamic equations. The iterative algorithm started with a guess at control efforts during the simulation time, then, using the Adams–Bashforth scheme, the states and co-states were calculated at each iteration; control efforts were then updated using the obtained states and co-states, and this continued until the difference in states and co-states in two sequential iterations became less than a predefined threshold.

Consider the following CF fractional differential equation:

(31) D0CFtαx(t)=f(t,x,u).      0<α≤1

where D0CFtα(.) is the CF fractional differential equation defined in [49]. By integrating Equation (31) using the CF fractional integral, we obtain:

(32) I0CFtα(D0CFtα(x(t)))=I0CFtα(f(t,x,u))

(33) $$x\left(t\right)-x\left(0\right)=\frac{1-\alpha }{M\left(\alpha \right)}f\left(t,x,u\right)+\frac{\alpha }{M\left(\alpha \right)}\int_{0}^{t}f\left(z,x\left(z\right),u\left(z\right)\right)dz$$

The time interval was discretized into steps with an interval of h; we thus have $t_{0}=0.\;t_{k+1}=t_{k}+h.\;\ldots .\;k=0:n-1$. Now, Equation (33) can be rewritten as

(34) $$x\left(t_{k+1}\right)-x\left(0\right)=\frac{1-\alpha }{M\left(\alpha \right)}f\left(t_{k},x\left(t_{k}\right),u\left(t_{k}\right)\right)+\frac{\alpha }{M\left(\alpha \right)}\int_{0}^{t_{k+1}}f\left(z,x\left(z\right),u\left(z\right)\right)dz$$

Also, we have

(35) $$x\left(t_{k}\right)-x\left(0\right)=\frac{1-\alpha }{M\left(\alpha \right)}f\left(t_{k-1},x\left(t_{k-1}\right),u\left(t_{k-1}\right)\right)+\frac{\alpha }{M\left(\alpha \right)}\int_{0}^{t_{k}}f\left(z,x\left(z\right),u\left(z\right)\right)dz$$

Subtracting Equation (35) from Equation (34) gives

(36) $$\begin{array}{cc} x\left(t_{k+1}\right)-x\left(t_{k}\right)= & \frac{1-\alpha }{M\left(\alpha \right)}\left(f\left(t_{k},x\left(t_{k}\right),u\left(t_{k}\right)\right)-f\left(t_{k-1},x\left(t_{k-1}\right),u\left(t_{k-1}\right)\right)\right)\\ & +\frac{\alpha }{M\left(\alpha \right)}\int_{t_{k}}^{t_{k+1}}f\left(t,x\left(t\right),u\left(t\right)\right)dt \end{array}$$

In order to calculate Equation (36), we approximated the integral $\int_{t_{k}}^{t_{k+1}}f\left(t,x\left(t\right),u\left(t\right)\right)dt$ by $\int_{t_{k}}^{t_{k+1}}K\left(t\right)dt$, where K(t) is a Lagrange interpolating polynomial of degree two that can be calculated using the following formula:

(37) $$K\left(t\right)=\overset{2}{\underset{i=0}{\sum }}f\left(t_{k-i},x\left(t_{k-i}\right),u\left(t_{k-i}\right)\right)L_{i}\left(t\right)$$

where the $L_{i}\left(z\right)$ terms are the Lagrange basis polynomials at each point. Using the aforementioned approximation, it can be proved that

(38) $$\begin{array}{cc} \int_{t_{k}}^{t_{k+1}}f\left(t,x\left(t\right),u\left(t\right)\right)dv= & h[\frac{23}{12}f\left(t_{k},x\left(t_{k}\right),u\left(t_{k}\right)\right)-\frac{4}{3}f\left(t_{k-1},x\left(t_{k-1}\right),u\left(t_{k-1}\right)\right)+\\ & \frac{5}{12}f\left(t_{k-2},x\left(t_{k-2}\right),u\left(t_{k-2}\right)\right)] \end{array}$$

where v is defined as $v=\frac{t_{k+1}-t}{h}$. Then, using Equation (38), the following recursive formula can be obtained for Equation (36):

(39) $$\begin{array}{cc} x\left(t_{k+1}\right)=x\left(t_{k}\right) & +\frac{1}{M\left(\alpha \right)}\left[\left(1-\alpha \right)+\frac{23}{12}h\alpha \right]f\left(t_{k},x\left(t_{k}\right),u\left(t_{k}\right)\right)\\ & -\frac{1}{M\left(\alpha \right)}\left[\left(1-\alpha \right)+\frac{4}{3}h\alpha \right]f\left(t_{k-1},x\left(t_{k-1}\right),u\left(t_{k-1}\right)\right)\\ & +\frac{5h\alpha }{12M\left(\alpha \right)}f\left(t_{k-2},x\left(t_{k-2}\right),u\left(t_{k-2}\right)\right) \end{array}$$

There is a truncation error for this approximation [38]. In order to find the solution of the fractional differential equation of the model Equation (24), we used Equation (39). During simulations, it was assumed that the order of all fractional derivatives was the same and was α=0.95. For simulating the fractional HIV/AIDS model, Equations (28) and (29) were written in vector form as follows:

(40) D0CFtαx(t)=f(t,x,u).      0<α<1

(41) D0CFtαλ(tf−t)=h(tf−t,x(tf−t),u(tf−t)).      0<α<1

Here,

(42) $$x\left(t\right)=\left[\begin{array}{c} S\left(t\right)\\ I\left(t\right)\\ \begin{array}{c} A\left(t\right)\\ T\left(t\right)\\ R\left(t\right) \end{array} \end{array}\right].f\left(t.x.u\right)=\left[\begin{array}{c} f_{1}\left(t,x,u\right)\\ f_{2}\left(t,x,u\right)\\ \begin{array}{c} f_{3}\left(t,x,u\right)\\ f_{4}\left(t,x,u\right)\\ f_{5}\left(t,x,u\right) \end{array} \end{array}\right].\lambda \left(t\right)=\left[\begin{array}{c} \lambda_{1}\left(t_{f}-t\right)\\ \lambda_{2}\left(t_{f}-t\right)\\ \begin{array}{c} \lambda_{3}\left(t_{f}-t\right)\\ \lambda_{4}\left(t_{f}-t\right)\\ \lambda_{5}\left(t_{f}-t\right) \end{array} \end{array}\right].h\left(t,x,u\right)=\left[\begin{array}{c} h_{1}\left(t_{f}-t,x,u\right)\\ h_{2}\left(t_{f}-t,x,u\right)\\ \begin{array}{c} h_{3}\left(t_{f}-t,x,u\right)\\ h_{4}\left(t_{f}-t,x,u\right)\\ h_{5}\left(t_{f}-t,x,u\right) \end{array} \end{array}\right]$$

Further, $f_{1}\left(t,x,u\right)=\Lambda -\beta \left(1-\varepsilon_{1}u_{1}\left(t\right)\right)I\left(t\right)S\left(t\right)-u_{3}\left(t\right)S\left(t\right)-dS\left(t\right)$, $f_{2}\left(t,x,u\right)=\beta \left(1-\varepsilon_{1}u_{1}\left(t\right)\right)I\left(t\right)S\left(t\right)+\varepsilon_{2}u_{2}\left(t\right)T\left(t\right)-dI\left(t\right)-k_{1}I\left(t\right)-k_{2}I\left(t\right)$, $f_{3}\left(t,x,u\right)=k_{1}I\left(t\right)-\left(\delta_{1}+d\right)A\left(t\right)+\alpha_{2}T\left(t\right)$, $f_{4}\left(t,x,u\right)=k_{2}I\left(t\right)-\varepsilon_{2}u_{2}\left(t\right)T\left(t\right)-\left(\alpha_{2}+d+\delta_{2}\right)T\left(t\right)$, and $f_{5}\left(t,x,u\right)=u_{3}\left(t\right)S\left(t\right)-dR\left(t\right)$. Moreover, co-state fractional differential equation vectors were defined as $h_{1}\left(t_{f}-t,x,u\right)=A+\beta \left(1-\varepsilon_{1}u_{1}\left(t_{f}-t\right)\right)I\left(t_{f}-t\right)\left(\lambda_{2}\left(t_{f}-t\right)-\lambda_{1}\left(t_{f}-t\right)\right)-\left(u_{3}\left(t_{f}-t\right)+d\right)\lambda_{1}\left(t_{f}-t\right)$, $h_{2}\left(t_{f}-t,x,u\right)=\beta \left(1-\varepsilon_{1}u_{1}\left(t_{f}-t\right)\right)S\left(t_{f}-t\right)\left(\lambda_{2}\left(t_{f}-t\right)-\lambda_{1}\left(t_{f}-t\right)\right)-\left(d+k_{1}+k_{2}\right)\lambda_{2}\left(t_{f}-t\right)+k_{1}\lambda_{3}\left(t_{f}-t\right)+k_{2}\lambda_{4}\left(t_{f}-t\right)$, $h_{3}\left(t_{f}-t,x,u\right)=-\left(\delta_{1}+d\right)\lambda_{3}\left(t_{f}-t\right)$, $h_{4}\left(t_{f}-t,x,u\right)=-\varepsilon_{2}u_{2}\left(t\right)\lambda_{2}\left(t_{f}-t\right)+\alpha_{2}\lambda_{3}\left(t_{f}-t\right)+\left(u_{2}\left(t_{f}-t\right)-\left(\alpha_{2}+d+\delta_{2}\right)\right)\lambda_{4}\left(t_{f}-t\right)+B$, and $h_{5}\left(t_{f}-t,x,u\right)=-d\lambda_{5}\left(t_{f}-t\right)$. Finally, using the recursive formula in Equation (39), the solution of both Equations (28) and (29) was obtained. The system parameters for the simulations are given in Table 1. The optimal controller parameters were considered as $A=20,B=300,C_{1}=1,C_{2}=20,\;\mathrm{and}\;C_{3}=1$. In addition, the fractional-order of the CF derivative was considered as α=0.98.

### 7.1. Strategy A: Control Using Treatment Alone

**Figure 1:**
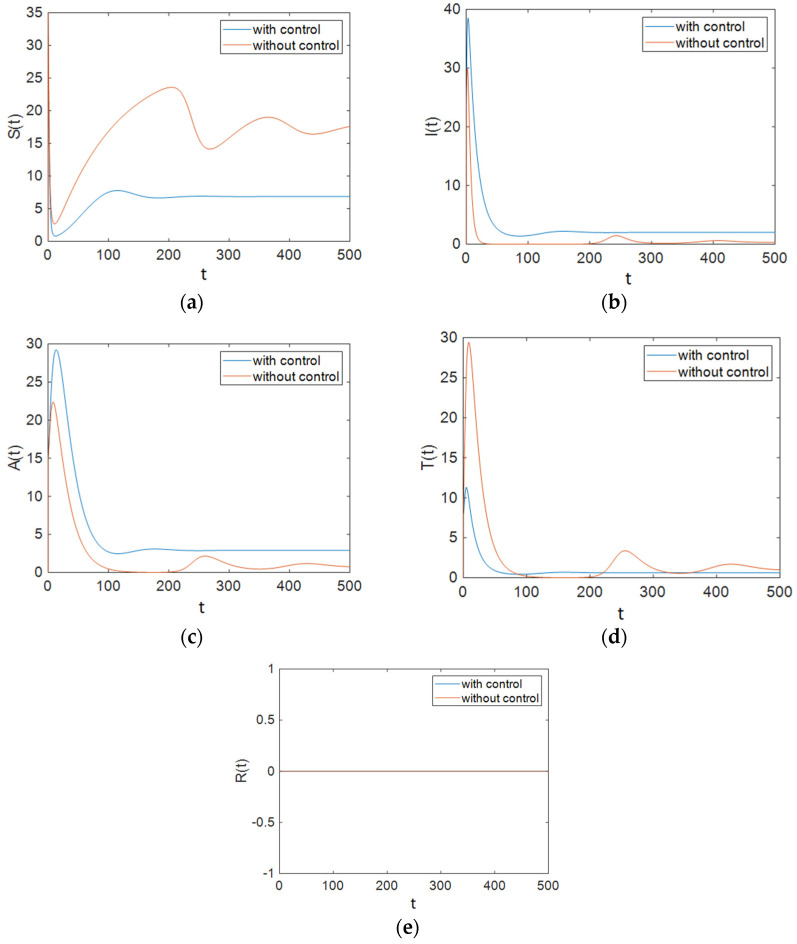
The number of individuals under Strategy A, which includes the use of ART: (**a**) Susceptible patients; (**b**) People who are infectious; (**c**) Individuals for whom the treatment is not effective; (**d**) Individuals being treated with ART and for whom the treatment is effective; (**e**) Individuals who have changed their sexual habits sufficiently.

In this strategy, only the control $u_{2}$ was used to control the ART rate in the treated compartment per unit time. Figure 1 shows the results of the simulation of the case when only the control $u_{2}$ was applied to the system. It can be seen that for the aim of minimizing the population of the treated compartment, this control effort performed well, but it had detrimental effects on other states.

**Figure 2:**
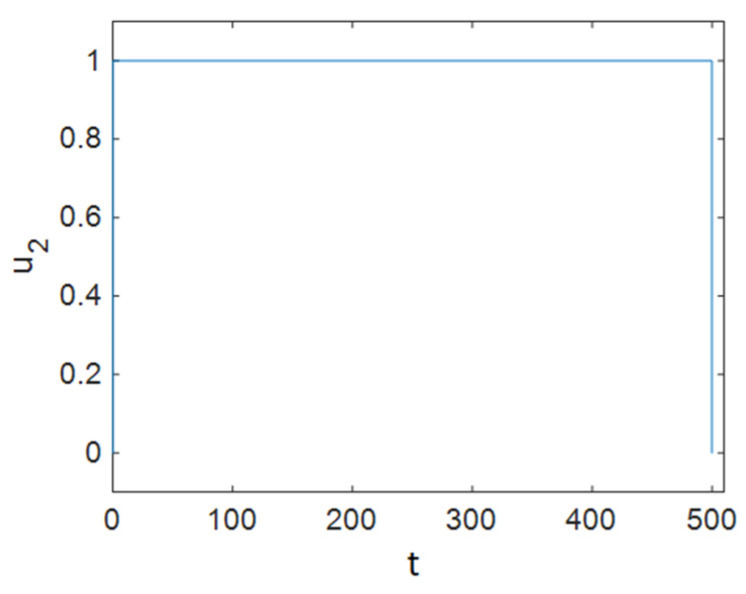
Time history of the optimal control effort $u_{2}$ (ART treatment).

**Figure 3:**
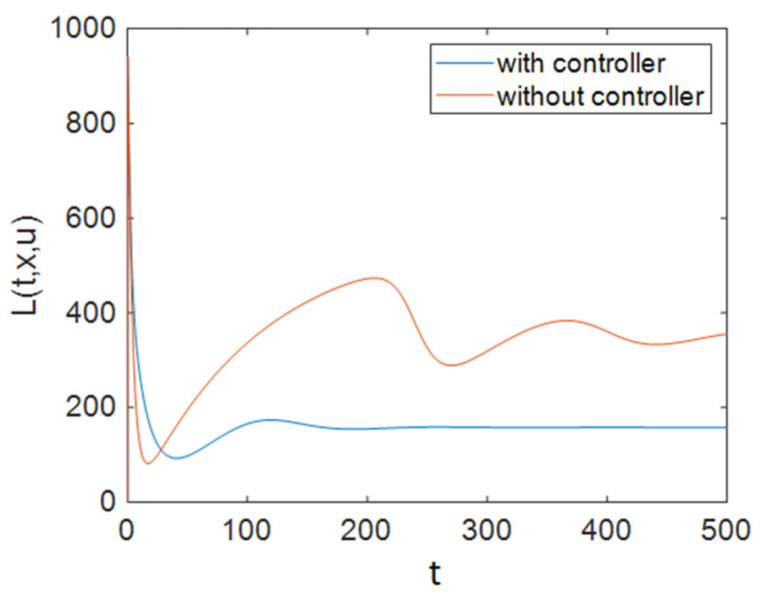
The time history of function L when applying Strategy A.

It can be seen in Figure 2 that control efforts were at 0 for approximately 100 days, and then they increased and reached 1; again, after a period of time, they returned to 0 linearly, and as is obvious, at the end of the simulation the control efforts $u_{1}$ had reached a zero value. It should be noted that for the simulations of the system without a controller, the values of control inputs were considered to be constant at $u_{1}=\frac{1}{\varepsilon_{1}},\;u_{2}=0,\;\mathrm{and}\;u_{3}=0$. Figure 3 depicts the time history of function L when Strategy A was applied to the system. Based on Figure 3, the optimal controller effectively reduced function L. As shown in Figure 1, this strategy (only the use of ART) could not improve the situation for all groups. Therefore, we need to apply prevention actions with ART, which are investigated in the next sections.

### 7.2. Strategy B: Control Using Treatment and Changes in People’s Sexual Habits

**Figure 4:**
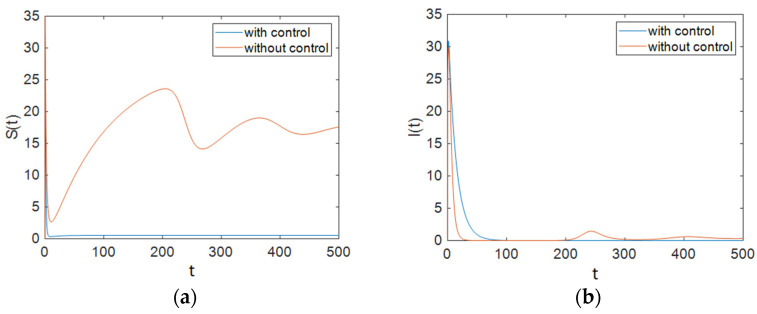

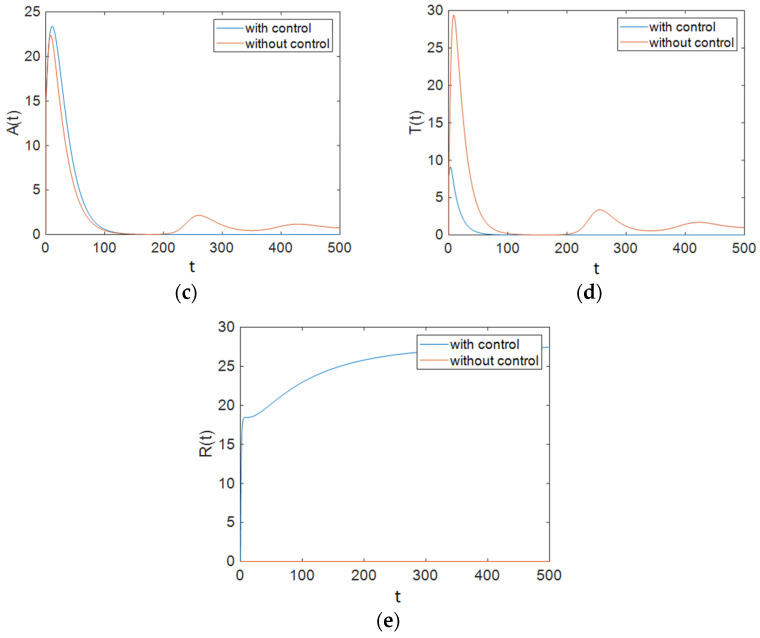
The number of individuals under Strategy B, which includes ART treatment and changes in the sexual habits of susceptible patients: (**a**) Susceptible patients; (**b**) People who are infectious; (**c**) Individuals for whom the treatment is not effective; (**d**) Individuals being treated with ART for whom the treatment is effective; (**e**) Individuals who have changed their sexual habits sufficiently.

In this section, the behavior of the system was simulated for the case in which $u_{2}$ and $u_{3}$ are used to control the ART rate in the treated compartment per unit time and the proportion of susceptible people who have changed their sexual habits per unit time. The simulation results show the effectiveness of controlling the aforementioned parameters. Figure 4 shows that the population of susceptible people decreased significantly compared to the case with no control effort on the system, and it also shows that the number of people with full-blown AIDS did not change greatly from that in the case with no control effort. However, it can be observed that the population of the treated compartment reached zero over time. Furthermore, it can be seen that the number of people in the removed compartment increased remarkably.

**Figure 5:**
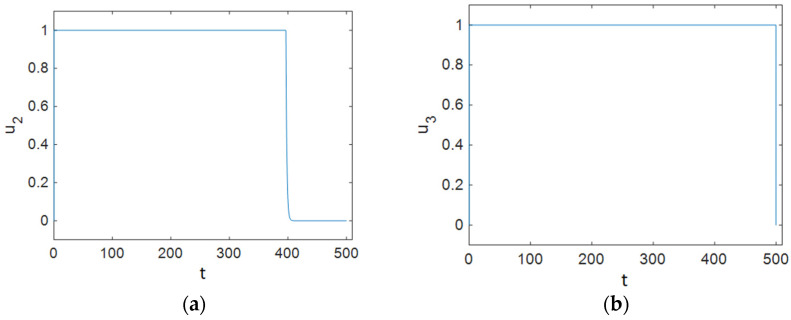
Time history of the optimal control efforts: (**a**) $u_{2}\;\left(\mathrm{ART}\;\mathrm{treatment}\right)$; (**b**) $u_{3}\;\left(\mathrm{changes}\;\mathrm{in}\;\mathrm{the}\;\mathrm{sexual}\;\mathrm{habits}\;\mathrm{of}\;\mathrm{susceptible}\;\mathrm{patients}\right).$

**Figure 6:**
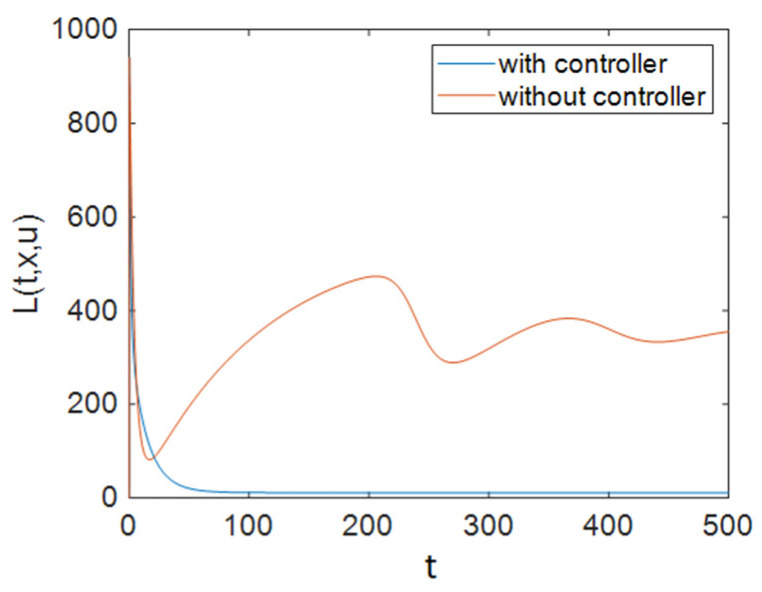
The time history of function L when applying Strategy B.

As Figure 5 shows, ART was applied to individuals in the treated compartment at its maximum rate for half of the simulation time, and it dropped and reached zero after about 40 days; it can be concluded that people in the susceptible compartment must maintain changes in their sexual habits for most of the time, and it can be seen that the control efforts *u*_3_ decreased sharply at the end of the simulation and reached zero. Additionally, Figure 6 demonstrates that Strategy B effectively decreased the value of function L.

### 7.3. Strategy C: Control Using Prevention, Treatment, and Changes in Sexual Habits

**Figure 7:**
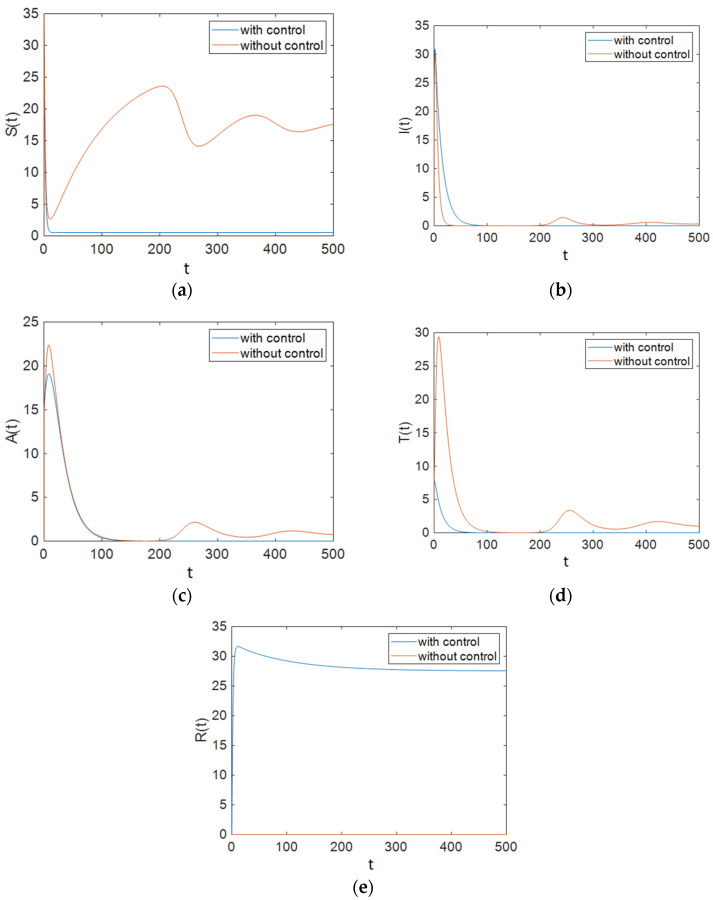
The number of individuals under Strategy C, which includes condom use, ART treatment, and changes in the sexual habits of susceptible patients: (**a**) Susceptible patients; (**b**) People who are infectious; (**c**) Individuals for whom the treatment is not effective; (**d**) Individuals being treated with ART for whom the treatment is effective; (**e**) Individuals who have changed their sexual habits sufficiently.

**Figure 8:**
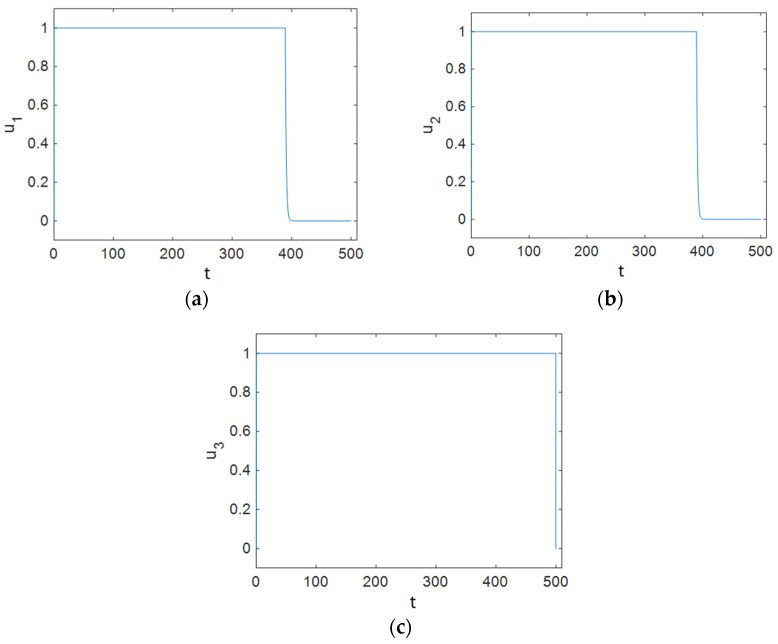
Time history of the optimal control efforts: (**a**) $u_{1}\;\left(\mathrm{condom}\;\mathrm{use}\right);$ (**b**) $u_{2}\;\left(\mathrm{ART}\;\mathrm{treatment}\right)$; (**c**) $u_{3}\;\left(\mathrm{changes}\;\mathrm{in}\;\mathrm{the}\;\mathrm{sexual}\;\mathrm{habits}\;\mathrm{of}\;\mathrm{susceptible}\;\mathrm{patients}\right).$

**Figure 9:**
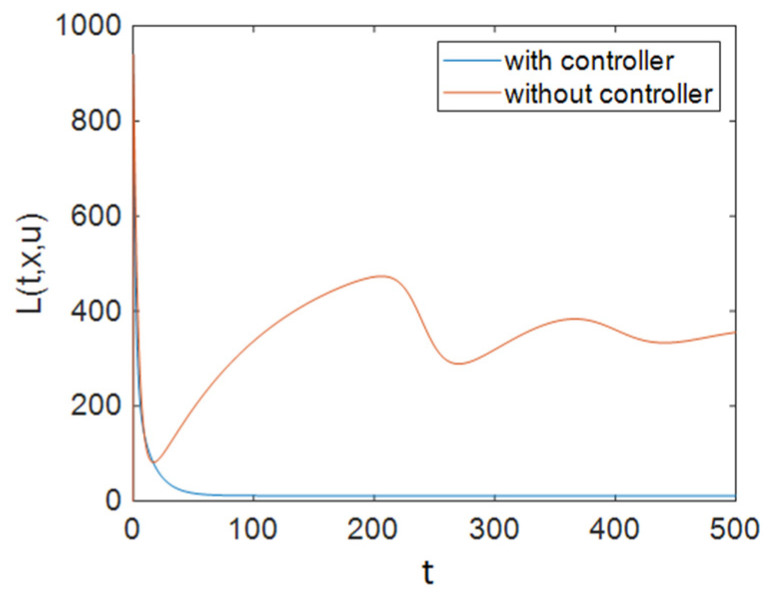
The time history of function L when applying Strategy C.

In this case, all of the control efforts were applied to the system in order to minimize the predefined cost function, Equation (20). The results of the simulation show that the population of the susceptible compartment plunged; it remained at a low level and did not rise again. Besides this, the maximum number of infected people decreased compared to that in the case with no control effort, and the rate of decrease in the population of infectious individuals was faster. In addition, Figure 7 shows that the number of individuals with full-blown AIDS reached zero faster than it did in the case with no control effort. As can be observed in Figure 7, the maximum number of people in the treated compartment declined significantly, and the population decreased more sharply than it did in the case where no control effort was applied to the system. Figure 8 shows the time history of control efforts; it can be seen that control effort $u_{1}$ was at 1 for about 400 days, then it decreased delicately and reached 0, so it can be concluded that the contact rate of susceptible people and individuals in the infectious class must decrease by the use of condoms at a minimum rate. In addition, it is obvious that the rate of ART plunged after about 400 days and then reached zero slowly, so after about 400 days, there is no need for ART. Figure 8 shows that the individuals in the susceptible people compartment must maintain changes in their sexual habits until the end of the simulated time period. Figure 9 shows that the value of function L was reduced by Strategy C.

### 7.4. Comparing Different Strategies

As shown in Figure 1, Strategy A, which only applies ART, could not improve all groups’ situations. In Strategy B, the situation was better. As demonstrated in Figure 4, the population of susceptible people decreased significantly compared to that under Strategy A. In this strategy, the population of the treated compartment reached zero. Strategy C was the best one. In this strategy, the number of individuals with full-blown AIDS reached zero faster than it did in other strategies (especially compared with Strategy A). As shown in Figure 7, the maximum number of people in the treated compartment declined significantly, and the population of recovered people increased.

Also, to compare the results of all strategies easily, we can investigate the cost functions. As shown in Figure 3, Figure 6 and Figure 9, the value of the cost function in Strategy A was greater than those in Strategies B and C. Thus, it can be concluded that Strategy A is less effective than the other two investigated strategies.

## 8. Conclusions

In this paper, a CF fractional HIV/AIDS model was studied. In order to find control strategies to control disease, a sensitivity analysis was conducted. The results of the sensitivity analyses show that three parameters are more effective than others in controlling the disease. Using the results of these analyses, a modified model was proposed. The necessary conditions for the optimal control of the disease using control of the contact rate of susceptible and infectious people, control of the ART rate of the treated compartment population, and, finally, control of the rate of changes in the sexual habits of susceptible people were derived. Using a three-step fractional Adams–Bashforth scheme, simulations for four strategies were conducted, and the results of the simulations show that the best strategy is to use all of the control efforts simultaneously. Besides this, the results of the simulations show that the populations of the treated compartment and susceptible people class decreased at a higher rate under control strategies than when there was no controller. Furthermore, the population of the removed class increased notably. By considering the simulation results, it can be concluded that the proposed optimal controller is effective in controlling the disease. As a future suggestion, the advantages of the CF derivative can be used in the modeling of other biological systems. Furthermore, the optimal controller designed in the current paper is given as an open-loop controller. Hence, in a future study, by developing the proposed controller into a closed-loop one, its performance can be enhanced in dealing with modeling errors, uncertainties, and external disturbances.

## Figures and Tables

**Table 1:** The system parameters [49].

Parameter	Description	Value
Λ	The recruitment of susceptible people into the population	0.55
β	The contact rate between susceptible and infectious people	0.03
d	The natural death rate	0.0196
$k_{1}$	The rate at which leave the infectious class and become individuals with full-blown AIDS	0.15
$k_{2}$	The rate at which people with HIV receive treatment	0.35
$\alpha_{1}$	The rate at which treated individuals leave this compartment and return to the infectious compartment	0.08
$\alpha_{2}$	The rate at which individuals in the treated compartment leave this class and enter the AIDS compartment	0.03
$\delta_{1}$	The disease-induced death rate for individuals of the AIDS compartment	0.0909
$\delta_{2}$	The disease-induced death rate for individuals of the treated compartment	0.0667
$\mu_{1}$	The rate at which susceptible people change their sexual habits	0.03

**Table 2:** Sensitivity indices of to the parameters of the model.

Parameter	Description	Sensitivity Index
Λ	The recruitment of susceptible individuals into the population	1
β	The contact rate between susceptible and infectious individuals	1
$\alpha_{1}$	The rate at which treated individuals leave this compartment and return to the infectious compartment	−0.7231
$\alpha_{2}$	The rate at which individuals in the treated compartment leave this class and enter the AIDS compartment	0.1865
$\delta_{1}$	The disease-induced death rate for individuals of the AIDS compartment	0.0
$\delta_{2}$	The disease-induced death rate for individuals of the treated compartment	0.4147
$\mu_{1}$	The rate at which susceptible people change their sexual habits	0.1333

**Table 3:** The sensitivity indices of the state variables at the endemic equilibrium point.

Parameter	$S^{*}$	$I^{*}$	$T^{*}$
Λ	0.0000	0.1237	0.1237
β	−1.0000	−0.8762	−0.8762
$\alpha_{1}$	0.7231	−0.0894	−0.4970
$\alpha_{2}$	−0.1865	0.0230	−0.1297
$\delta_{1}$	0.0000	0.0000	0.0000
$\delta_{2}$	−0.4147	0.0513	−0.2885
$\mu_{1}$	−0.7382	0.6213	0.6213

## Data Availability

Not applicable.
